# A Data Fusion Approach to Enhance Association Study in Epilepsy

**DOI:** 10.1371/journal.pone.0164940

**Published:** 2016-12-16

**Authors:** Simone Marini, Ivan Limongelli, Ettore Rizzo, Alberto Malovini, Edoardo Errichiello, Annalisa Vetro, Tan Da, Orsetta Zuffardi, Riccardo Bellazzi

**Affiliations:** 1 Bioinformatics Center, Institute for Chemical Research, Kyoto University, Kyoto, Japan; 2 Department of Electrical, Computer and Biomedical Engineering, University of Pavia, Pavia, Italy; 3 Genomic Core Center, IRCCS Fondazione San Matteo, Pavia, Italy; 4 enGenome S.r.l., Via Ferrata 5, Pavia, Italy; 5 Centre for Health Technologies, University of Pavia, Pavia, Italy; 6 IRCCS Fondazione S. Maugeri, Pavia, Italy; 7 Department of Molecular Medicine, University of Pavia, Pavia, Italy; University of Catanzaro, ITALY

## Abstract

Among the scientific challenges posed by complex diseases with a strong genetic component, two stand out. One is unveiling the role of rare and common genetic variants; the other is the design of classification models to improve clinical diagnosis and predictive models for prognosis and personalized therapies. In this paper, we present a data fusion framework merging gene, domain, pathway and protein-protein interaction data related to a next generation sequencing epilepsy gene panel. Our method allows integrating association information from multiple genomic sources and aims at highlighting the set of common and rare variants that are capable to trigger the occurrence of a complex disease. When compared to other approaches, our method shows better performances in classifying patients affected by epilepsy.

## Introduction

Genome wide association studies (GWAS) have been used to test associations between common genomic variants, typically defined as those having a population frequency of 1% or higher, and complex diseases [1–3]. However, it has been shown that both common and rare (or novel) variants can independently or cumulatively contribute to complex traits [4–10]. Nowadays, the dropping costs of next generation sequencing technologies (NGS) allow directly assaying rare variants and sequencing a number of samples large enough to reach the statistical power requested by association studies exploiting common variants [11, 12]. Rare variants, however, suffer from both an increased multiple testing burden (due to large numbers and lower linkage disequilibrium) and a limited statistical power, due to their low frequencies [13]. Burden (or collapsing) and nonburden tests have been developed to address these issues [14–16]. Burden tests are more suitable in case of the assumption that all genomic variants are causal and with the same direction effect on the phenotype, while nonburden tests are more powerful when variant effects can be in different, even opposite, directions [17–19]. In order to maximize the power in sequenced-based association studies, methods to best combine these two approaches have been developed as well [4, 20]. All these methods combine genomic variants within single units, or regions of interest (ROIs), that are tested as single elements for association with the target phenotype. Typically, one considers as ROIs the genomic regions codifying for proteins, i.e. genes. However, several studies have expanded the concept of ROI beyond the genome, associating them to functional units. For example, Miller and coworkers showed that defining ROIs on the basis of conserved protein domains can effectively improve a gene-burden association test in cancer-sequenced genomes [21]; Wu and his team reported that pathway-based association methods can achieve higher power than gene-based methods on exome sequencing data in chronic obstructive pulmonary diseases [22] and protein-protein interactions (PPIs) have been used to study epistatic SNPs interactions in genotype-based GWAS in atherosclerosis [4, 23].

Recently, it has been shown that mining information from different data sources to discover new disease-gene-networks [24] and derive prediction models is a promising approach [25]. Predictive classifiers using genetic sequencing data are very important applications for clinical diagnosis of common and complex diseases [26, 27]. However, integrating heterogeneous data in presence of noise and uncertainty can be a serious challenge.

We hereby propose a data fusion-based burden method, combining common and rare genomic variants identified by NGS technology along genes, conserved domains, pathways and protein-protein interactions.

This method has been evaluated on sequencing data obtained by a panel of 109 genes that are involved in epileptic disorders in a cohort of 85 individuals aged 1-4 years, showing heterogeneous epilepsy phenotypes, all with seizures onset since birth or early infancy, and compare the data with 61 controls. The evaluation has been performed through a binary classification model (epilectic/non-epilectic) and by the use of ROIs as model features. Epilepsy is a symptom of different and heterogeneous conditions, affecting about 1% of the population, with different levels of penetrance and expressivity, frequently associated with comorbidities such as intellectual disability, and psychiatric disorders. [28, 29]. Common variants can only have a minor role in causing epilepsy [30], whereas rare, or even individual, variants, possibly play a major one [31]. Finally, since traditional GWA studies left epileptogenesis largely unexplained even considering large cohorts of patients [30, 32, 33], sequencing data have been proposed as a viable alternative to overcome the typical GWAS limitations [34–36].

In our results we have found that the capability to distinguish between epileptic patients and controls on genetic bases increases when different ROIs categories, rather than a single one (e.g. genes), are taken into account, and targets of association discovery are broadened. Finally, the ROIs that resulted more important for patients classification are significantly more mutated in cases than in controls, matching previous findings [37–40].

## Materials and Methods

### Framework

Our objective was to augment the feature space of association studies, traditionally limited to genes only, by integrating protein interactions, domains and pathways. This allowed the design of more powerful phenotype predictive models (discriminating cases from controls), and the test for disease association along four different axes.

Genomic variants were extracted and collapsed over the different ROIs for each patient. The features are encoded by the output of the collapsing method. We used the derived features for both univariate and multivariate analysis, spanning over the four types of ROIs.

**Fig 1:**
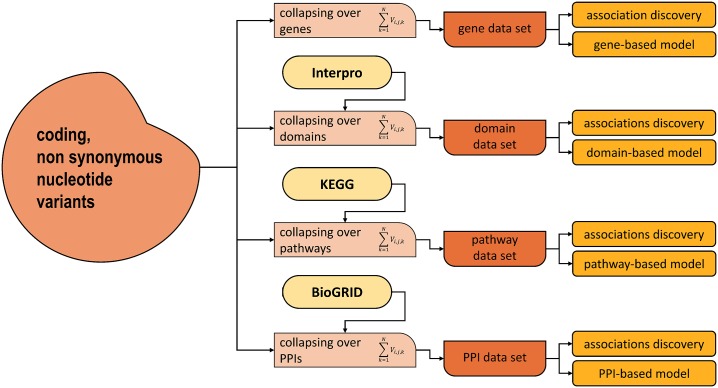
Data fusion framework. Sequencing data are collapsed to calculate their mutational loads using four ROIs, namely genes, pathways, domains and PPIs. This allows studying ROI-phenotype associations along the four correspondent axes. Each element tested for association then becomes a feature for a prediction model. Single ROI types are combined to create data sets. Each data set is split into a training and test set. The training set is used to tune the learning parameters of a RF model and then select the best set of features, while the test set is used to measure the prediction performances.

The different ROI types were combined to assemble fifteen data sets, such as genes + pathways, or domains + PPIs. Each data set was split into a training and a test set, used to train and test a Random Forest (RF) model. Fig 1 depicts the learning and testing workflow.

Our work is based on the following steps: (A) data acquisition and preprocessing, (B) univariate analysis, (C) multivariate predictive modeling. Note that the univariate analysis and multivariate predictive modeling are run independently.

1. data acquisition and preprocessing: Use NGS techniques to map case and controls genomic variants. Collapse variants along ROIs: genes, domains, pathways and PPIs. Assemble the data sets, one for each ROI type and eleven as ROI type combinations.
2. Perform univariate analysis on each ROI type to find statistically relevant elements. Measure p-values of each element.
3. Perform multivariate predictive modeling. Repeat 10 times the following and average the results: i) randomly split each data set into a training and a test set; ii) Learn a prediction model from the training set and use it to predict the labels of the test set. Measure average MCC and AUC score on the test sets.

### Data preprocessing

The data set exploited to assess our method is made of 146 samples, corresponding to 85 individuals with different types of either isolated or syndromic epileptic disorders and 61 controls.

Epilepsy patients were diagnosed by a multidisciplinary team. They were Caucasian 90 subjects, suffering from idiopathic epilepsy [generalized epilepsy with febrile seizures plus (GEFS+), 91 severe myoclonic epilepsy of infancy (Dravet syndrome), myoclonic astatic 92 epilepsy (MAE), benign familial neonatal seizures (BFNS)]or epileptic encephalopathies [pyridoxine-dependant epilepsy 94 (two cases), Lennox-Gastaut syndrome (two cases), and Ohtahara syndrome (one case)].

Epilepsy cases had 109 genes sequenced according to an extension of the gene panel proposed by Della Mina et al. [37]. The list of genes included in the panel is available in the Supporting Information. Controls are made of 15 healthy individuals sequenced by using the aforementioned gene panel and 61 whole-exome sequenced individuals (Agilent SureSelect Human All Exon v5).

Samples have been sequenced by Genome Analyzer IIx and generated reads have been filtered according to base quality within the first 32 bases. BWA software v0.6.0 has been used to map reads against GRCh37/hg19 genomic reference and Samtools v0.1.18 to filter out potential PCR duplicates. Reads were recalled by base quality and those overlapping known and common genetic indels were realigned more accurately by GATK v.1.9 software. Single nucleotide and indels variants have been identified by GATK Unified Genotyper within the solely genomic regions belonging to the 109 selected genes and were filtered according to several quality rules such as the proportion of not-uniquely mapped reads (>10%), reads mapping quality by depth (>5), strand bias, variant quality (>50, Phred scale) and variant coverage (>8x).

Controls consisted of 15 voluntary blood donors sequenced by using the aforementioned gene panel and 46 whole-exome sequenced individuals (Agilent SureSelect Human All Exon v5) selected among healthy parents and relatives of probands suffering from Fanconi anemia, Blackfan Diamond anemia, short stature, developmental sex disorders. All of them were Caucasian.

Genomic variants have been annotated by ANNOVAR [41], by using NCBI RefSeq as gene track and 1000 Genome Project (1TGP) for variant frequencies. We enriched the ANNOVAR output by considering protein domains overlapping the variants from InterPro [42]; gene-related pathways from KEGG [43]; and genes grouped by the physical protein-protein interactions (PPIs) among them, recorded in BioGRID [44]. The details of feature encoding are explained in next Section.

### ROIs and collapsing method

Our data set is focused on genes known to be associated with different epileptic phenotypes, therefore a large fraction of variants in a genomic region might have both causal and risk-increasing effects (same direction and magnitude). As consequence, a burden test was selected as collapsing method.

The variants are collapsed into four ROIs: genes, domains, pathways and PPIs. To this end, we modified the approach proposed by Tatonetti and coworkers [45] by including the information on the presence of one or two mutated alleles for a genomic locus (heterozygosity or homozygosity) and the predicted mutation impact on protein stability, used as a mutation weight. This approach penalizes the contribution of common variants to the final score but also limits the weight of rare variants that might be tolerated for proteins and therefore have a neutral effect over the phenotype. For each *ROI*_*j*_, and for each individual *i*, a ROI score is assigned with the following additive model:

$$\begin{array}{c} \sum_{k=1}^{N}v_{i,j,k} \end{array}$$ (1)

$$\begin{array}{c} -v_{i,j,k}=\sigma_{i,j,k}\times P_{i,j,k}\times \log\left(F_{V_{i,j,k}}\right) \end{array}$$ (2)

Where *N*_*j*_ is the number of variants overlapping *ROI*_*j*_, and *k* is a single variant. *P* is the variant score computed by PaPI [46], an algorithm predicting the mutational load of a given variant. (PaPI score varies between 0 and 1, with 1 being the maximum confidence that the given variant is deleterious.) *σ* has three possible values, 0, 1, or 2, depending on the number of variant alleles (no variant, heterogeneous or homogeneous respectively). *F*_*V*_*i*,*j*,*k*__ is the variant frequency in 1TGP data base.

Each variant therefore contributes to each ROI score per individual. Note that a single variant can contribute to the scores of different ROIs. For example, a variant *v* overlaps the domain *D* of the gene *A*; a gene *A* belongs to pathways *P*1 and *P*2 and also interacts with gene *B* and gene *C*. Therefore, variant *v* contributes to 6 ROIs: 1 gene (gene *A*), 1 domain (*D*), 2 pathways (*P*1 and *P*2) and 2 genes involved in PPIs (genes *B* and *C*).

**Fig 2:**
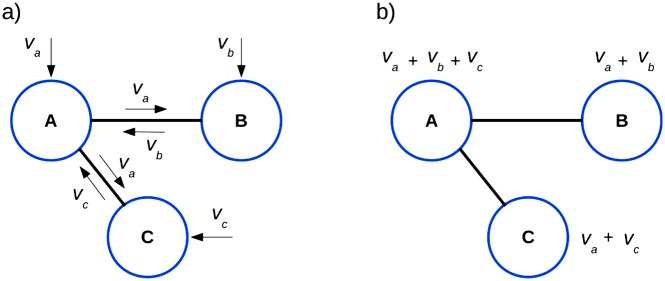
Examples of variants influencing more than one gene. Protein encoded by gene *A* interacts with proteins encoded by gene *B* and gene *C*. (a) Variant on gene *A*, *V*_*A*_ contributes to the score both for *A* and *B* due to their interaction. In the same way, variants on gene *B*, *V*_*B*_, and on gene *C*, (*V*_*C*_) both contribute to the scores of genes *A* *B* and *C*. (b) Resulting variant contributions on final PPIs scores.

Fig 2 shows how gene variants contribute to ROI scores by propagating mutational load to interacting genes at protein level (PPIs).

### Data sets

Four initial data sets are generated by each of the different ROI group (252 domains as InterPro domains overlapping gene coding regions; 129 genes as RefSeq annotated genes, including isoforms; 130 pathways as KEGG pathways related to the gene panel and 569 genes referred from physical PPIs in BioGRID). By combining them, we obtained 15 different data sets.

### Univariate analysis

In the univariate analysis we performed either student T test or Wilcoxon signed rank test (in case of non-Gaussian distribution) with Bonferroni correction to discover ROIs associated with epilepsy. The selected ROIs were then analyzed in terms of their interconnections in case they share variants, as described above.

### Multivariate analysis—Learning

As a learning strategy, we applied RF as implemented in the Weka framework [47]. RF is an ensemble method widely used in Bioinformatics. Note that, in order to avoid overfitting, the results of the univariate analysis obtained on the whole data set were ignored. Multi- and univariate analysis were run independently. To reduce the high number of features (up to 252 + 129 + 130 + 569 = 1080, when all ROI types are considered), feature selection was applied with the Binary outcome stochastic search algorithm(BOSS) [48]. BOSS is a Bayesian model selection method ranking features based on their probability of inclusion in the prediction models obtained by a Markov Chain Monte Carlo iterative approach. It showed promising results in SNP selection [49]. According to previous works [48, 49], we set our prior model size to be 20, with a variance of 10; and for each run, the total iterations were 100 thousands, including 10 thousands for the burn-in phase.

Data were randomly divided into training (70%) and test (30%) set. We considered as parameters to be optimized: (a) the number of selected features (5, 10, 25, 50, 100 and unselected features), and (b) the number of trees (5, 10, 50, 100, 150, 200, 250, 300, 350, 400, 450, 500). As depicted in Fig 1, parameters, including the number of selected features, were optimized with a 5-fold cross validation on the training sets (all other parameters were left at default value) to avoid overfitting. Feature selection was performed independently on each fold of the training sets, and test data were not involved in the process. The best parameter combination in the training cross validation was retained and utilized to predict the test set. Area under ROC curve (AUC) and Matthews Correlation Coefficient (MCC) were calculated. The whole process was repeated 10 times, and the results averaged.

## Results

### Univariate-analysis: data fusion allows performing gene, domain, pathway and protein interaction association studies

**Fig 3:**
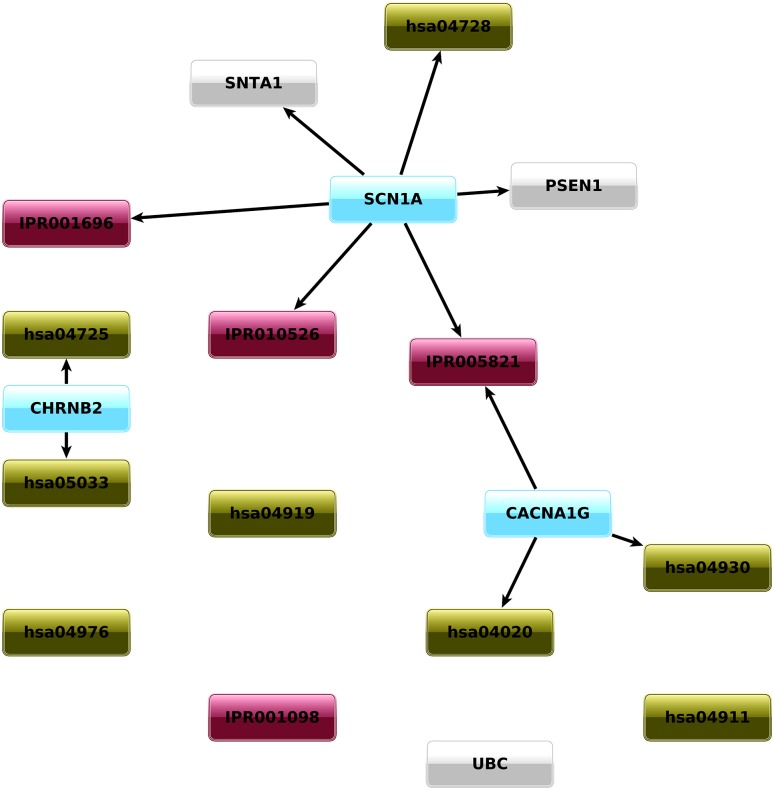
Interconnected, statistically significant ROIs. Graphical representation of statistically significant ROIs and their overlapping. The direction of the arrow means that an element is included into another. Gene ROIs (light blue) can be part of pathway (green) or PPI (grey) ROIs, while domain ROIs (purple) can be part of gene ROIs.

By considering four ROI types, we assess the phenotype association of genes, domains, pathways and PPIs. The univariate analysis on the four axes found three genes, four domains, eight pathways and three PPIs to be significantly related to epilepsy, as depicted in Fig 3 and reported in Tables 1 and 2. Note that, thanks to the data fusion approach, the intersection between the different ROIs emerges (for example, we found the four associated genes to be included in five of the associated pathways, as shown in Table 2).

**Table 1:** Genes associated with the phenotype.

ROI	Element	p-value
	SCN1A	5.83 × 10^−6^
genes	CACNA1G	6.56 × 10^−5^
	CHRNB2	3.04 × 10^−5^

p-values and associated genes of gene ROIs.

**Table 2:** Domains, pathways and PPIs associated with the phenotype.

ROI	Element	p-value	related genes
	IPR001098	1.63 × 10^−4^	POLG
Domains	IPR001696	3.56 × 10^−5^	**SCN1A**, SCN2A, SCN9A
	IPR010526	2.99 × 10^−5^	**SCN1A**, SCN2A, SCN9A
	IPR005821	9.59 × 10^−6^	CACNA1A, **CACNA1G**, CACNA1H, KCNA1, KCNMA1, KCNQ2, KCNQ3, **SCN1A**, SCN2A, SCN9A
	hsa04930	1.2 × 10^−6^	CACNA1A, **CACNA1G**, SLC2A2
Pathways	hsa04725	7.99 × 10^−5^	CACNA1A, CHRNA4, **CHRNB2**, KCNQ2, KCNQ3, PLCB1
	hsa04728	1.18 × 10^−6^	CACNA1A, GRIN2A, GRIN2B, PLCB1, PPP2R2C, **SCN1A**, SLC6A3
	hsa05033	1.42 × 10^−5^	CACNA1A, CHRNA4, **CHRNB2**, GABRA1, GABRB3, GABRD, GABRG2, GRIN2A, GRIN2B
	hsa04020	4.22 × 10^−6^	CACNA1A, **CACNA1G**, CACNA1H, GRIN2A, PLCB1, PTK2B
	hsa04911	1.55 × 10^−5^	ATP1A2, KCNMA1, KCNN3, PLCB1, SLC2A1, SLC2A2
	hsa04919	2.71 × 10^−4^	ATP1A2, NOTCH3, PLCB1, SLC2A1
	hsa04976	1.75 × 10^−4^	ATP1A2, SLC2A1
PPIs	UBC	1.44 × 10^−5^	FLNA, TUBA1A, UBE3A, SLC9A6, SLC1A3, PAFAH1B1, STXBP1, GPR56, NEDD4L, SLC2A1, ASPM, FANCI, TIMM17B, KCTD7, PLCB1, PDCD10, OPA1, NOTCH3, SCARB2, SLC25A22, HTT, DYRK1A, ATP6AP2, ALDH7A1, DMD, PQBP1, ARAF, TUBB2B, CSTB, TAP1, PTK2B, MECP2, TBC1D24, CNTNAP2, GRIN2A, CLCN2, PPP2R2C, POLG, STRADA, GABRD, CACNA1A, ATP1A2, MEF2C, KCNN3, SLC4A10, GABRB3, RBFOX1, JRK, PRICKLE1, TCF4, ARHGEF9, GRIN2B, SLC6A3, CASR, NHLRC1, EPM2A, OPRM1, GABBR1, CLN8
	SNTA1	4.24 × 10^−6^	DMD, **SCN1A**, KCNJ10, SLC6A3
	PSEN1	5.83 × 10^−6^	**SCN1A**

p-values and related genes of domain, pathway and PPI ROIs. Related genes associated to the phenotype are in bold font.

### Multivariate analysis—More accurate models are obtained by integrating different data sources

The averaged AUC scores of the multivariate models calculated on the independent test sets are shown in Table 3.

**Table 3:** Classification performance for each data set.

data set	AUC (feat. selection)	AUC (unselected feat.)	MCC (feat. selection)	MCC (unselected feat.)
genes	0.86	0.83	0.55	0.48
domains	0.76	0.82	0.55	0.54
pathways	0.77	0.76	0.37	0.43
PPIs	0.81	0.79	0.44	0.38
**genes + domains**	**0.89**	**0.87**	**0.64**	0.57
genes + pathways	0.84	0.83	0.5	0.52
genes + PPIs	0.84	0.79	0.49	0.42
**domains + pathways**	0.88	0.86	0.54	**0.58**
domains + PPIs	0.87	0.81	0.59	0.45
pathways + PPIs	0.82	0.8	0.47	0.45
**genes + domains + pathways**	**0.89**	0.86	0.6	0.56
genes + pathways + PPIs	0.85	0.8	0.53	0.43
genes + domains + PPIs	0.87	0.83	0.56	0.52
domains + pathways + PPIs	0.88	0.82	0.6	0.48
genes + domains + pathways + PPIs	0.86	0.83	0.53	0.52

Performance of prediction models on the test sets averaged on ten repetitions. The highest value of each column is bold. Feature selection increases AUC in 14 out of 15 data sets up to +6%, and MCC in 12 out of 15, up to +12%.

The results show that augmenting the feature space by including ROIs other than genes leads to better results. Our baseline is a traditional approach solely based on unselected genes features (0.83 AUC, 0.48 MCC). Compared to it, several feature combinations are superior in terms of AUC, MCC, or both. For example, genes + domains (0.87 AUC, 0.57 MCC), domains (0.54 MCC), genes + domains (0.87 AUC, 0.57 MCC), genes + pathways (0.52 AUC), domains + pathways (0.58 AUC). After feature selection, the performances increase up to 0.89 AUC (genes + domains; genes + domains + pathways) and 0.64 MCC (genes + domains). Feature selection has a positive effect on most of the data sets, especially when the data have a high number of initial features. For example, the performance on the PPI only data set (569 features) has an increase of 2% in AUC and of 6% in MCC after feature selection. In two cases, however, when the initial features are lower, the performance worsens: this happens in the pathway only (129 features, +1% AUC, −6% MCC) and in the domains only (-6% AUC, +1%MCC) data sets.

The combination of genes + domains has the best results. Pathways, however, demonstrate to be a viable feature representation, and when added to domains show the highest MCC (0.58) on unselected features. Moreover, in terms of AUC, while domains and pathways taken alone perform worse than genes (0.82 and 0.76 vs 0.86, respectively), once combined they perform better (0.88 vs 0.86). PPIs, on the other hand, do not play a pivotal role in feature representation. They increase the performances only when added to domains and pathways, but they have a lower impact than adding genes features. With reference to the way the PPI features are encoded, we argue that their representation redundancy burdened the signal carried by genes. We speculate that PPIs can be better exploited when a wider gene panel is available, or perhaps when whole genome sequencing data is used.

### Power analysis

Effects of the sample size on the models performances have been studied for both association study (univariate analysis) and prediction models (multivariate analysis). As univariate analysis is concerned, the power is higher than 0.8 for all the ROIs that have been found statistically significant. Results have been obtained following the approach proposed in [50], by: (i) simulating new data based on the variables distribution observed in cases and controls (mean, standard deviation, number of observations); (ii) estimating the Cohen’s size effect; and (iii) performing a Wilcoxon Rank Sum test to derive statistically significant differences in terms of the simulated variables distribution between class = 1 and class = 0 to compute the corresponding p-value. More details about this procedure, as well as the power analysis graphs, are provided in the Supporting Information.

Multivariate power analysis has been determined via learning curves calculation [51]. In particular, this method consists of iteratively sampling a growing subset of individuals from a data set and measure the power. We considered sample sizes in the range (5, 6, 7, 8, 9, 10, 20, 30, 40, 50, 60, 70, 80, 90, 100, 110, 120, 130, 140, 145, 146), the latter being the full data set. The power has been calculated by averaging a hundred 5-fold cross validation for each step. Learning curves of the different data sets have been calculated with a model based on the parameters retained for test classification, i.e. the parameters of the learning curves were not optimized for the task, thus representing a lower bound for power (sensitivity) analysis. As expected, learning curves tend to grow with the number of samples, plateauing mostly around 120 to 135 samples, where the 95% confidence intervals overlap with the maximum sensitivity. Sensitivity ranges between 0.7 and 0.73. Confidence intervals range around 0.05, with less than ten samples, to about 0.01 with a hundred samples or more. Only three sets require the whole set to reach full power, namely the genes only data set, the PPIs only data set, and their combination genes + PPIs data set. This is in accordance with the prediction results, where PPIs failed to provide increased performances. Sample size analysis suggests that adding or considering ROI types other than PPIs leads to a more stable sensitivity, requiring less samples to plateau. Graphs of the learning curves are reported in the Supporting Information.

## Discussion

In this paper, we showed how genomic data fusion (i.e. the combination of different data source in genomics) can be utilized to designed more accurate prediction models and elucidate associations in complex diseases.

### Intersecting results from gene, domain, pathway and PPI features

Thanks to the data fusion approach, we were able to compare results on different ROIs pairwise.

#### Genes

All the three significant genes we found in our study are ion-channel-related genes. CACNA1G and SCN1A are calcium-channel and sodium-channel related genes respectively, while CHRNB2 is a ligand-gated ion channel gene. These three genes are all expressed in nervous system tissues (Table A in S1 File) during embryogenesis, but also at later stage of brain development. Given our gene panel, this was expected. The result indeed confirmed that channelogenesis plays an important role in sporadic or syndromic epilepsy [37, 39, 40, 52]. We did not identify any gene other than channel-related genes relating to epilepsy.

#### Domains

Four domains were found to be significant: IPR001098, IPR001696, IPR010526 and IPR005821.

While IPR001098 has a significant p-value, interestingly, its corresponding gene (POLG) is not included in the list of significant genes. POLG is a gene encoding the catalytic subunit of mitochondrial DNA polymerase gamma and is susceptible in children with intractable epilepsy; it comprises three main domains: an N-terminal domain containing 3′ → 5′ exonuclease activity, a spacer domain and C-terminal domain, containing 5′ → 3′ DNA polymerase activity in three subdomains termed palm, fingers and thumb [53]. The palm domain (IPR001098) is the one shown significant in our study. The mutational loads of other domains of POLG are not relevant in data.

IPR001696 is related to voltage gated sodium channel, IPR010526 to sodium ion transport, and IPR005821 is found in sodium, potassium, and calcium ion channels proteins. These results are consistent with the role of channelopathy in epileptogenesis [39, 54].

Both IPR001696 and IPR010526 belong to three genes in the gene panel: SCN1A, SCN2A and SCN9A; and both of them are subunits of SCN1A that is, as previously discussed, a significant gene in our panel. However, IPR001696 and IPR010526 both present a higher p-value when compared to SCN1A (3.56 × 10^−5^ and 2.29 × 10^−5^ vs 5.83 × 10^−6^). We argue that the two domains contribute as causative factors to the part SCN1A plays in epileptogenesis.

IPR005821 is a domain in ten genes of the considered sequencing panel: CACNA1A, CACNA1G, CACNA1H, KCNA1, KCNMA1, KCNQ2, KCNQ3, SCN1A, SCN2A, SCN9A. Among them CACNA1G and SCN1A are those mainly significantly mutated. While our analysis suggests an implication of IPR005821 in epileptogenesis, further analysis are needed to clarify the possible involvement of the other genes it belongs.

#### Pathways

hsa04725, hsa04728, hsa0503, and hsa04020 have at least one significant gene in their corresponding gene list Table 2. Besides has04725, all the other pathways features are more significant than their corresponding significant gene. For example, has04728 contains the significant gene SCN1A, whose p-value is higher than hsa04728 (5.83 × 10^−6^ vs 1.18 × 10^−6^). This means that all these pathways contains other genes that contribute to epilepsy. Interestingly, CACN1A is in the list of all pathway-corresponding genes. This strongly implies that CACN1A plays a role in epileptogenesis, even if it has not been included in the gene features. In contrast, hsa04725 contains CACNA1A and CHRNB2, but its p-value is higher than CHRNB2 alone (7.99 × 10^−5^ vs 3.04 × 10^−5^). This may suggest that, although CACNA1A contributes to epilepsy mutational load, the noise from other pathway-related genes (CHRNA4, KCNQ2, KCNQ3 and PLCB1) compromises this effect. These results confirms that, although it is not among the significant gene list, CACNA1A has a role in epilepsy.

The pathway hsa04930 includes a significant gene (CACNA1G). The larger CACNA1G p-value (6.56 × 10^−5^) when compared to hsa04930 p-value (1.2 × 10^−6^) suggests that the other two genes CACNA1A and/or SLC2A2 may contribute to epilepsy with small effect sizes. CACNA1A is a Calcium Channel, Voltage-Dependent, P/Q Type, Alpha 1A Subunit; SLC2A2 is a solute carrier family 2 (Facilitated Glucose Transporter); hsa04930 is pathway related to type 2 diabetes. Although there are evidences that seizures are common in hyperglycemia and are often the first manifestation [55], it is still not entirely clear that this pathway is directly linked with epilepsy.

There are other three significant pathways with no significant genes included: hsa04976, hsa04919 and hsa04911. They nonetheless share some common genes which weight indicates susceptible genetic factors. Pathway hsa04976 contains only ATP1A2, SLC2A1, while hsa04919 contains the same two genes, plus NOTCH3 and PLCB1. Since the p-value of hsa04919 (2.71 × 10^−4^) is higher than the one of hsa04976 (1.74 × 10^−4^), NOTCH3 and PLCB1 might carry noise. On the other hand, pathway hsa04911 carries the same two genes of hsa04976, plus KCNMA1, KCNN3, PLCB1, SLC2A1, SLC2A2. Excluding PLCB1 (contained in hsa04919), this gene group can embed some small signal. Further analysis conducted on ATP1A2, SLC2A1, SLC2A2 KCNMA1 and KCNN3 showed they all are related to epilepsy or seizures [38, 39, 56, 57].

The significant pathways have a very heterogeneous spectrum in terms of functions as reported in KEGG database: hsa04725 is a Cholinergic synapse pathway, hsa04020 is a calcium signaling pathway, hsa04930 is a type 2 diabetes-related pathway, hsa04976 is a bile secretion pathway, hsa04728 is involved in dopaminergic synapses, has05033 is involved in nicotine addiction; hsa04911 is a insulin secretion pathway; and hsa04919 is a thyroid hormone signaling pathway. hsa04976 and hsa04919 pathways are not directly linked to epilepsy.

In Supporting Information Table B in S1 File, reports a more detailed description of diseases associated pathways.

#### PPIs

From a PPI perspective, SNTA1 PPI features showed a significant p-value. SNTA1 group contains four genes belonging to our gene panel: DMD, SCN1A, KCNJ10, SLC6A3, among which SCN1A is a significant gene in our gene feature association test. The higher p-value of SCN1A (5.83 × 10^−6^), compared to SNT1A PPI (4.24 × 10^−6^), may suggest small mutational loads from DMD, KCNJ10 and/or SLC6A3 probably play a role in the association. While KCNJ10 and SLC6A3 codify for ion channels and thus their contribution to epileptogenesis is not unexpected, DMD codes for dystrophin, which has been traditionally associated with X-linked Duchenne and Becker muscular dystrophies. Nevertheless, a few reports supported its potential role in seizure generation as well [58].

UBC PPI feature includes 59 genes in our gene panel, although none of them appeared to be significant when tested alone for association. This suggests that those 59 genes, taken together, have a significant association signal with epilepsy.

As the third PPI feature PSEN1 is concerned, we found only one correspondence in the significant genes list (SCNA1). Therefore the p-value from this PPI feature is a replicate of SCNA1.

### Limitations of the approach

A major limitation of our association study is the number of candidate genes. We argue that by using a broader list of candidate genes, our approach of pair-wise comparison of association signals of different collapsed ROIs can be more useful for unraveling the genetic factors of complex diseases. However, as the number of candidate genes increases, the data noise will increase as well. Therefore, feature optimization and noise control before the association discovery might be helpful.

Another limitation is related to the variants weighing scheme of the model: PaPI weights only coding variants by predicting its deleteriousness on the protein product, and excluding the variants in the non-coding regions of genes. This may lead to the loss of causative variants in non-coding regions of the candidate genes, including splicing mutations.

Finally, it is worth noting that the selection of healthy parents and relatives of probands suffering from serious genetic diseases might have introduced a bias on the study.

## Conclusions

We have presented a data fusion framework able to merge different genomic features (called ROIs) in terms of gene, domain, pathway and protein-protein interaction data by using genomic variants identified in NGS experiments. Our framework well suites burden association test and aims at exploiting both common and rare variants in the assessment of a complex disease diagnosis through a classification model trained on a dataset of cases and controls.

We tested our method on a dataset of epileptic (85) and healthy individuals (61) that have been sequenced according to a target gene panel composed by 109 epilepsy-related genes. We then applied the framework in order to compute the mutational load for each ROI starting by genomic variants identified by NGS. We finally tested the association for each ROI in cases and controls and built a classification model to discover the best combination of ROIs for our dataset.

It is not surprising that the ROI elements found significantly associated to epilepsy have been previously found to be causative, because our candidate gene panel was derived from literature review. However, we found that some ROIs are significantly associated to the disease even if their related genes are not, as in the case of POLG gene (not significant) and its domain IPR001098 (significant) or the hsa04911 pathway (significant) and its related genes (not significant). Moreover, the only three genes that independently were found to be significantly mutated in cases with respect to controls confirmed that channelopathy plays a very important role in epilepsy.

We finally built a classification model based on RF and tested different ROIs categories. The RF models provided a very promising result, showing the feasibility of integrating genes, domains, pathways and protein-protein interaction data in a predictive model. The predictive accuracy on a independent test set reached the AUC and MCC scores of, respectively, 0.89 and 0.64 with the “genes + domains” set of ROIs.

Our positive results are consistent with the hypothesis of shared genetic factors across different types of epilepsy [59] and provide more evidence that multiple variants within a gene or gene-domains, rather than single deleterious variants, may interplay to determine epilepsy susceptibility, potentially accounting for the incomplete penetrance reported for pathogenic variants in confirmed epilepsy genes [37, 40, 60]. We speculate that with larger sample size, more genetic factors of epilepsy will be included and the predictive accuracy of our model will be improved. Moreover, this approach can be tested and generalized for broader utilizations in other common diseases. It can be useful, for example, to screen susceptible genetic factors in common disease association studies by collapsing methods. In fact, in presence of NGS data and binary class, such as good/bad prognosis, or positive/negative outcome of a therapy, our method can be applied to train prediction models based on the genetic background of the single patient, including cancer, when somatic cell genomic information is available.

## Supporting Information

S1 File Supporting Information file. Here are the notes on the expression of the associated genes (Table A); the relevant disorders associated to the associated pathways (Table B); the list of genes utilized in the panel; and the details of the multivariate power analysis on every data set.
(PDF)
