# Supplementary material for: A Data Fusion Approach to Enhance Association Study in Epilepsy
Source: PLoS One. 2016 Dec 16;11(12):e0164940. doi: 10.1371/journal.pone.0164940 (PMC5161322; doi:10.1371/journal.pone.0164940)
Supplement: S1 File — Here are the notes on the expression of the associated genes (Table A); the relevant disorders associated to the associated pathways (Table B); the list of genes utilized in the panel; and the details of the multivariate power analysis on every data set. (PDF) [file pone.0164940.s001.pdf]

Supporting Information

“A data fusion framework to enhance association study in epilepsy”,

Simone Marini, Ivan Limongelli, Ettore Rizzo, Alberto Malovini, Edoardo Errichiello, Annalisa Vetro, Tan Da, Orsetta Zuffardi, Riccardo Bellazzi

Supplementary Table A	Page 2
Supplementary Table B	Page 3
List of genes utilized in the panel	Page 4
Sample size analysis, multivariate	Page 9
Sample size analysis, univariate	Page 24
Supplementary Bibliography	Page 27

Supplementary Table A

Notes on the expression of the associated genes (source: GeneCards)

Name	*Embryogenesis expression in nervous tissues*	
**SCN1A**	Brain (Nervous System)	Forebrain White Matter
**CACNA1G**	Brain (Nervous System)	Hypothalamus<br>Hippocampus<br>Thalamus<br>Cerebellum<br>Amygdala
**CHRNB2**	Neural Tube (Nervous System)	Primitive Spinal Cord<br>Telencephalon<br>Diencephalon<br>Metencephalic Alar Plate<br>Metencephalic Basal Plate
	Brain (Nervous System)	Pituitary Gland<br>Cerebellum<br>Striatum<br>Midbrain tegmentum

Supplementary Table B

Relevant disorders associated to the associated pathways (source: KEGG)

Name	Description	*Relevant associated KEGG diseases*
hsa04725	Cholinergic synapse - Homo sapiens (human)	Periodic paralysis<br><br>Early infantile epileptic encephalopathy<br><br>Hypokalemic periodic paralysis<br><br>Familial or sporadic hemiplegic migraine<br><br>Benign familial neonatal and infantile epilepsies<br><br>Autosomal dominant nocturnal frontal lobe epilepsy
hsa04728	Dopaminergic synapse - Homo sapiens (human)	Syndromic X-linked mental retardation with epilepsy or seizures<br><br>Familial or sporadic hemiplegic migraine<br><br>Febrile seizures<br><br>Obsessive-compulsive disorder
hsa04020	Calcium signaling pathway - Homo sapiens (human)	Familial or sporadic hemiplegic migraine<br><br>Neuromuscular disorders (such as Brugada syndrome, Hypokalemic periodic paralysis, Catecholaminergic polymorphic ventricular tachycardia, Brody myopath, Multi-minicore disease)
hsa04976	Bile secretion - Homo sapiens (human)	GLUT1 deficiency syndrome (resulting in hypoglycorrhachia. Affected individuals present with mental retardation and learning disabilities; also common are ataxia, dystonia, *seizures*, and acquired microcephaly)
hsa04911	Insulin secretion - Homo sapiens (human)	Defects in the degradation of ganglioside (resulting in the accumulation of undegraded substrates in neurons and skeletal tissues)
hsa04919	Thyroid hormone signaling pathway - Homo sapiens (human)	--
hsa05033	Nicotine addiction - Homo sapiens (human)	--
hsa04930	Type II diabetes mellitus - Homo sapiens (human)	--

List of genes utilized in the panel

ALDH7A1

ARAF

ARHGEF9

ARX

ASPM

ATP1A2

BRD2

CACNA1A

CACNA1G

CACNA1G-AS1

CACNA1H

CACNB4

CCM2

CDKL5

CEND1

CHRNA2

CHRNA4

CHRNB2

CLCN2

CLN8

CNTNAP2

CSTB

DCX

DKFZp686K1684

DMD

DYRK1A

EFHC1

EPM2A

FANCI

FLNA

FOXG1

GABBR1

GABRA1

GABRA6

GABRD

GABRG2

GJD2

GPR56

GPR98

GRIK1-AS2

GRIN2A

GRIN2B

HCN1

HTT

HTT-AS1

IPCEF1

JRK

KCNA1

KCNAB2

KCND2

KCNJ10

KCNMA1

KCNN3

KCNQ2

KCNQ3

KCTD7

KRIT1

LGI1

LOC100507463

LOC729683

MAGI2

MECP2

MIR3911

MIR548F3

MIR548I4

MIR548T

MLLT3

NDP

NEDD4L

NHLRC1

NOTCH3

OPA1

OPHN1

OPRM1

PAFAH1B1

PAX6

PCDH19

PDCD10

PDYN

PLCB1

POLG

PORCN

PPP2R2C

PQBP1

PRICKLE1

PSMB9

PTK2B

RBFOX1

RELN

RS1

SCARB2

SCN1A

SCN1B

SCN2A

SCN9A

SERPINI1

SHANK3

SLC1A3

SLC25A22

SLC2A1

SLC2A2

SLC4A10

SLC4A3

SRPX2

ST3GAL5

STRADA

STXBP1

SYN

SYP

TAP1

TBC1D24

TCF4

TIMM17B

TPSG1

TUBA1A

UBE3A

Sample size analysis, multivariate

Multivariate sample size analysis have been calculated via learning curves for each data set, measured for 5, 6, 7, 8, 9, 10, 20 … 140, 145, 146 samples. For each sample bin, sensitivity has been averaged over 100 repetitions of a 5 fold cross validation. The cutoff line represents sensitivity with the full data set (146 samples), while the red area is the confidence interval at 95%.

Supplementary Figures S1-S15 depict the learning curves for all the data sets.

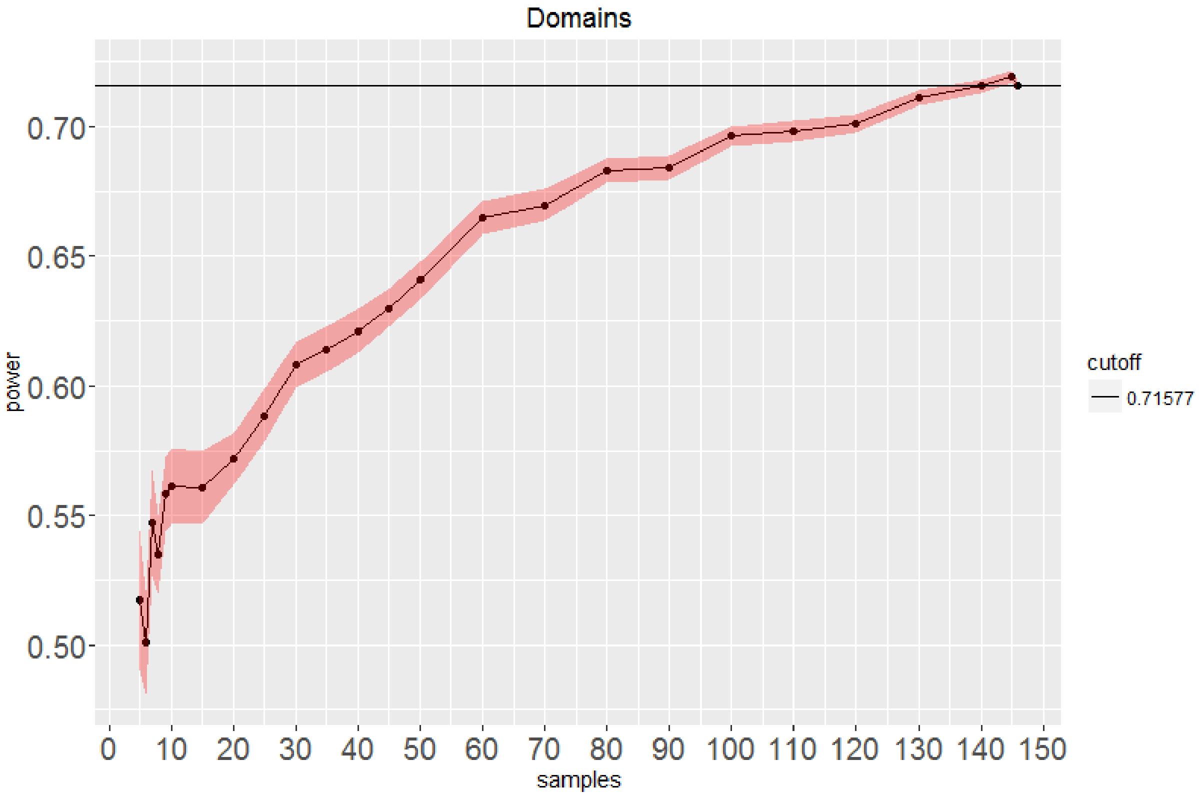

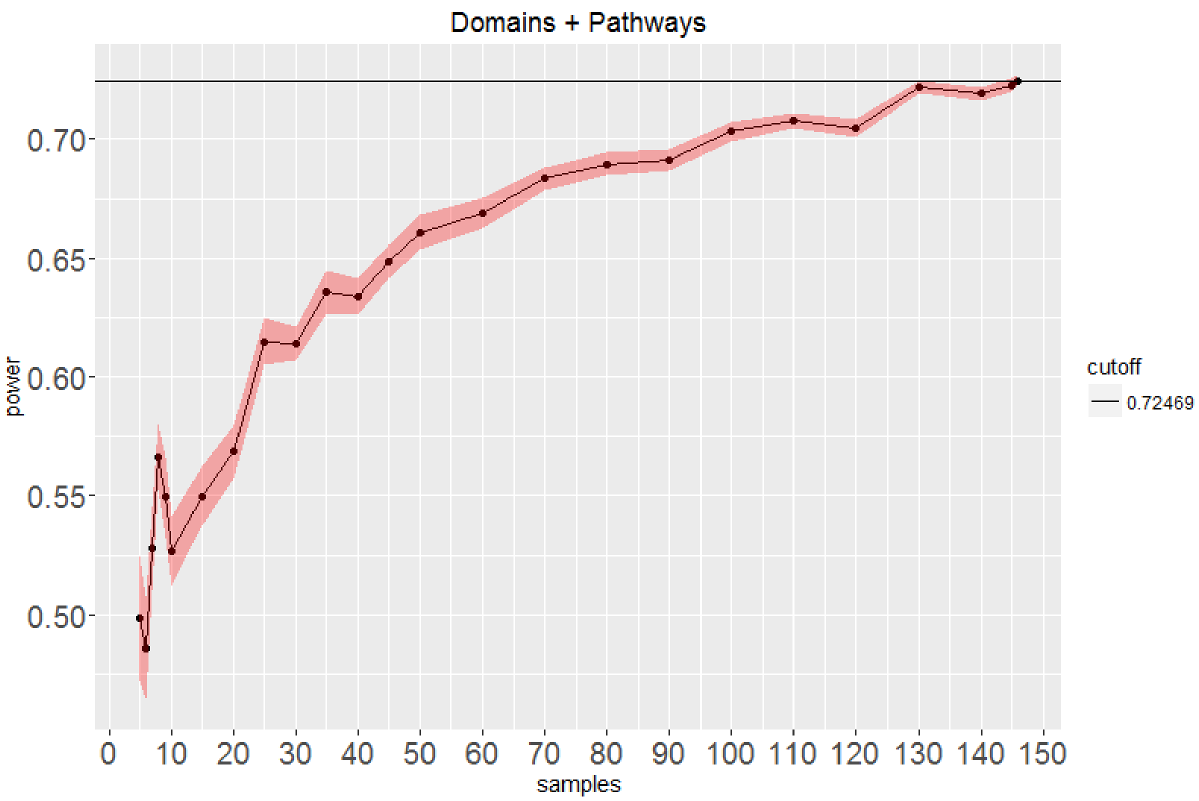
Domains + Pathways
power
0.50
0.55
0.60
0.65
0.70
samples
0
10
20
30
40
50
60
70
80
90
100
110
120
130
140
150
cutoff
0.72469

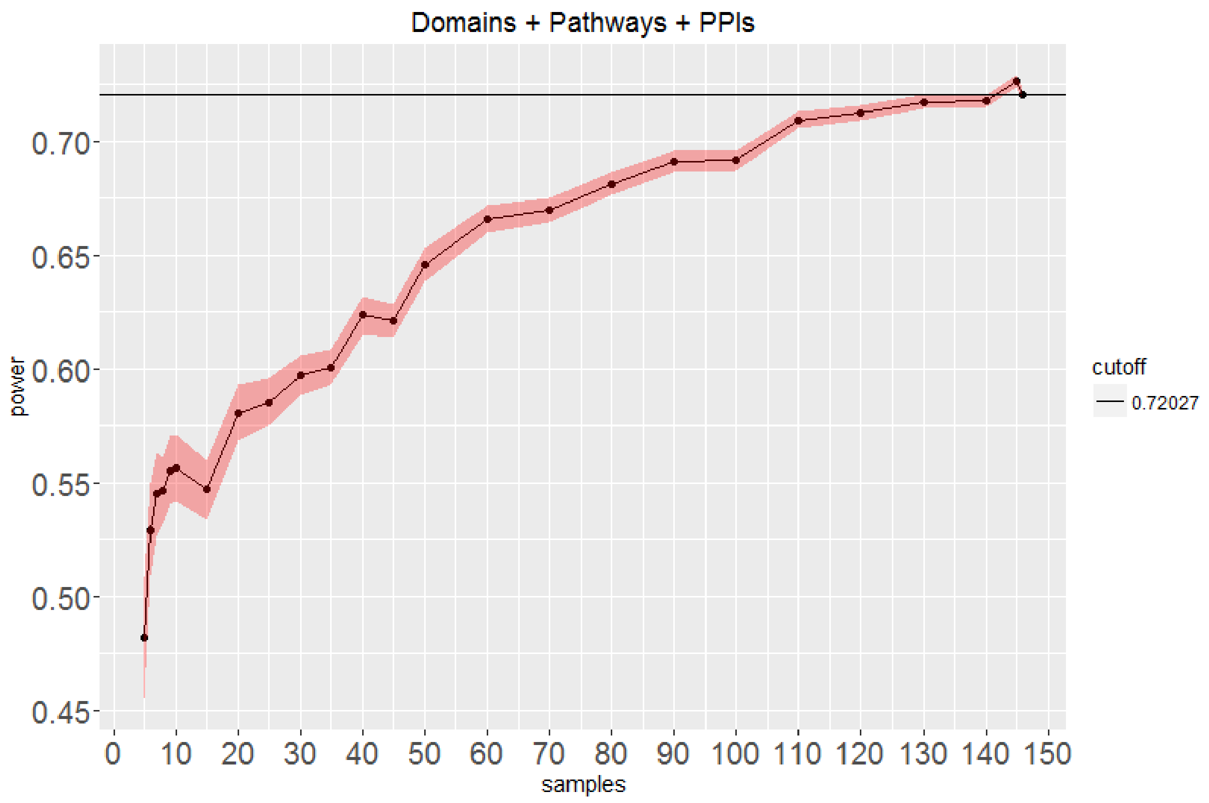
Domains + Pathways + PPIs
power
samples
0.45
0.50
0.55
0.60
0.65
0.70
0
10
20
30
40
50
60
70
80
90
100
110
120
130
140
150
cutoff
0.72027

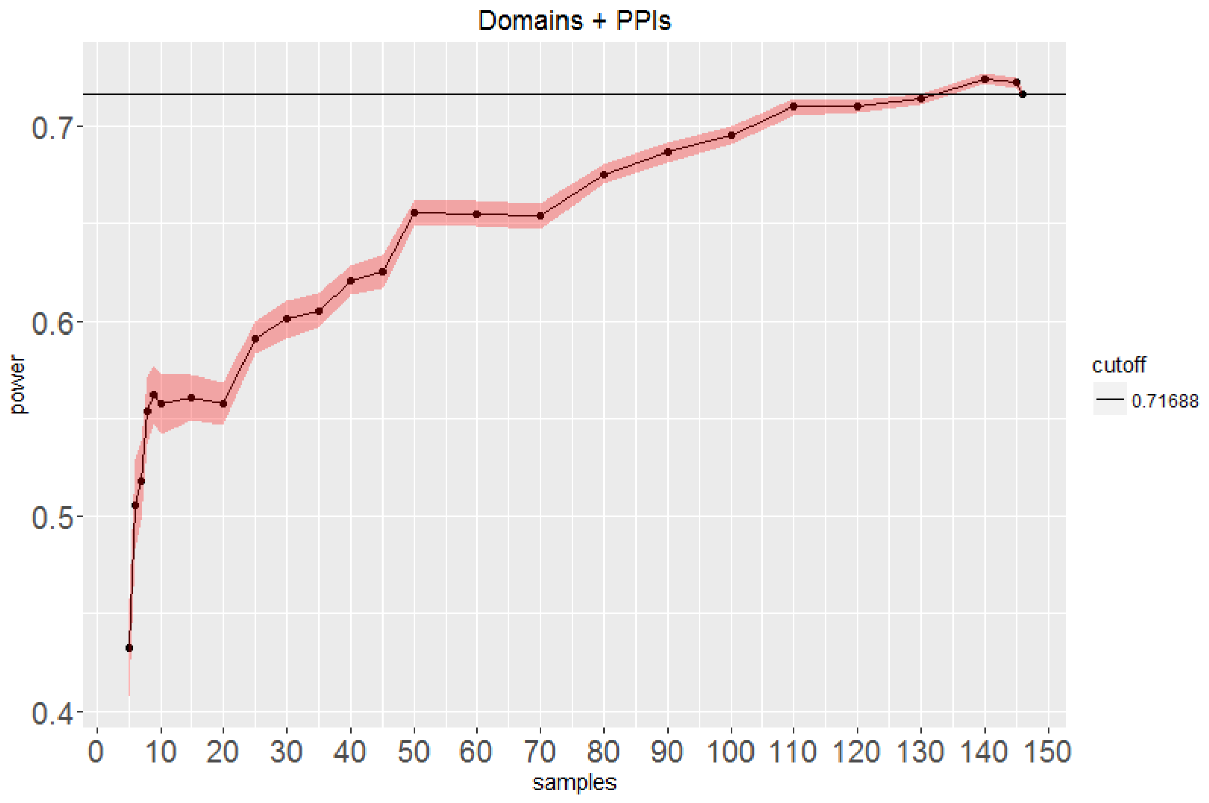

Domains + PPIs
power
samples
0.4
0.5
0.6
0.7
0
10
20
30
40
50
60
70
80
90
100
110
120
130
140
150
cutoff
0.71688

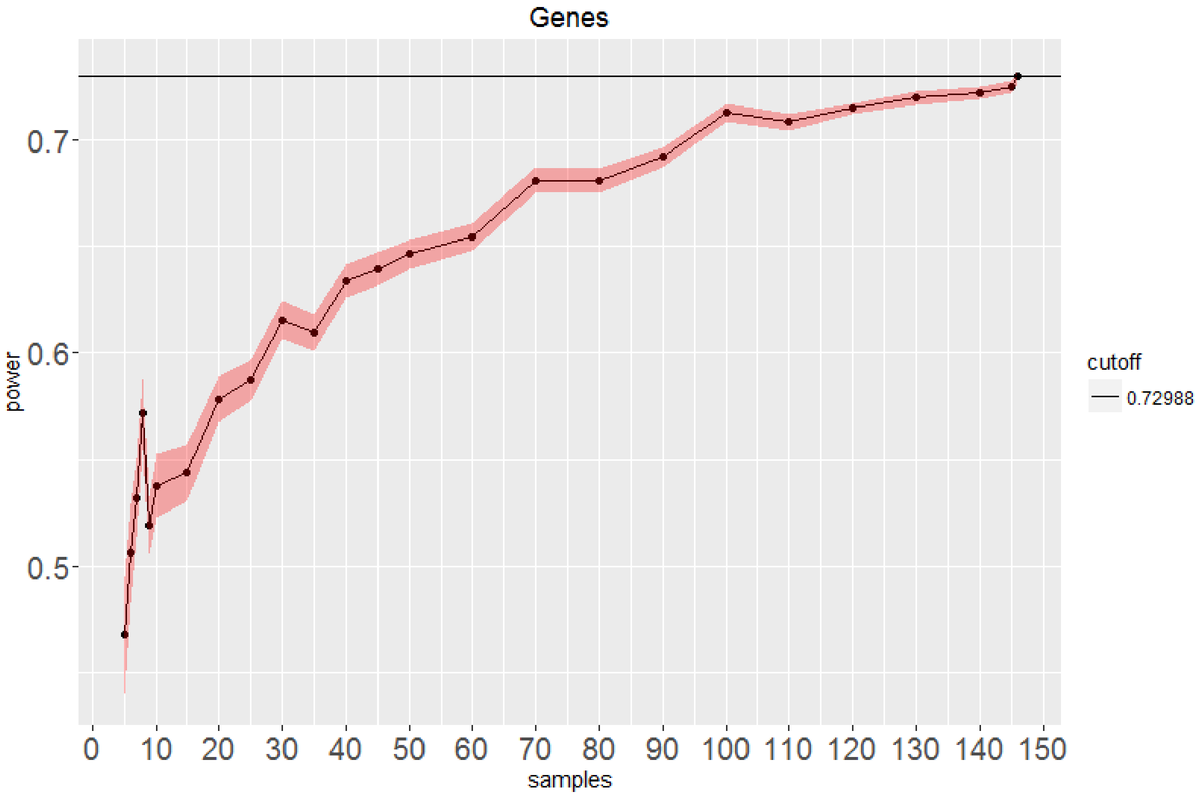

Genes
0.7
0.6
0.5
power
0 10 20 30 40 50 60 70 80 90 100 110 120 130 140 150
samples
cutoff
0.72988

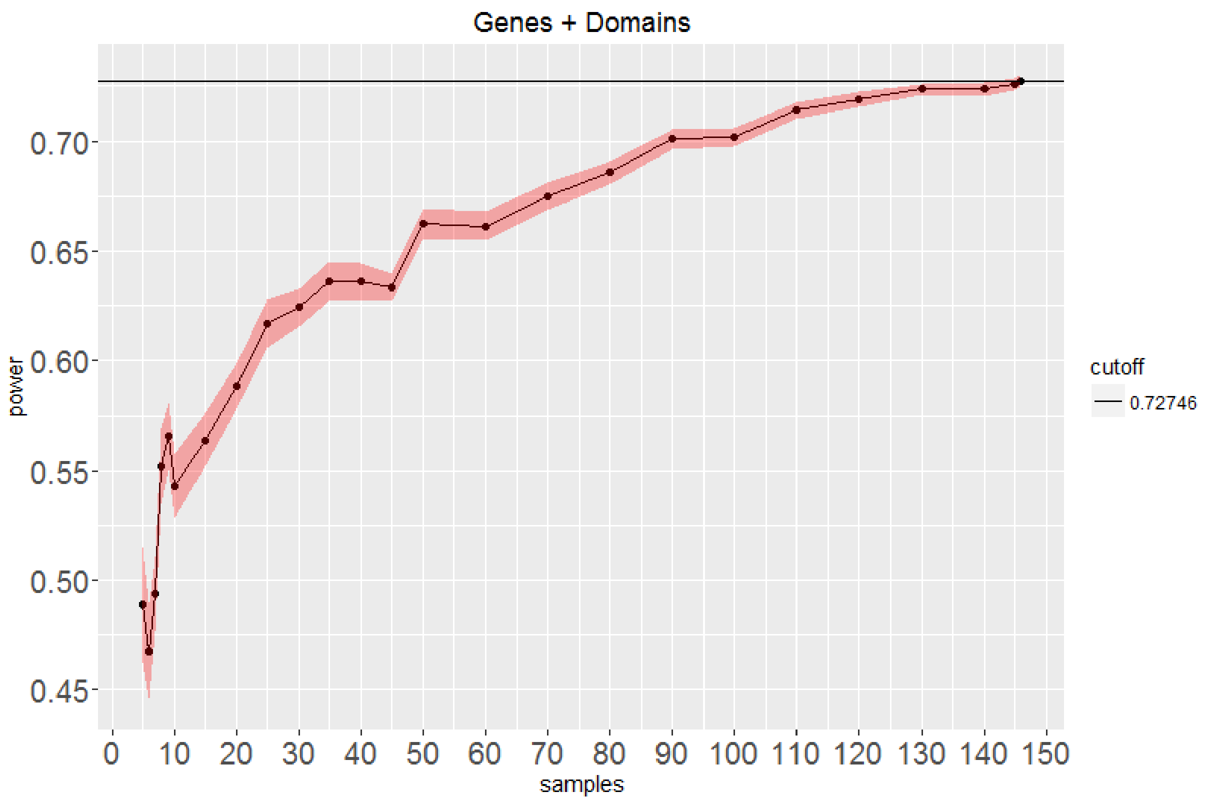
Genes + Domains
0.70
0.65
0.60
0.55
0.50
0.45
power
0
10
20
30
40
50
60
70
80
90
100
110
120
130
140
150
samples
cutoff
0.72746

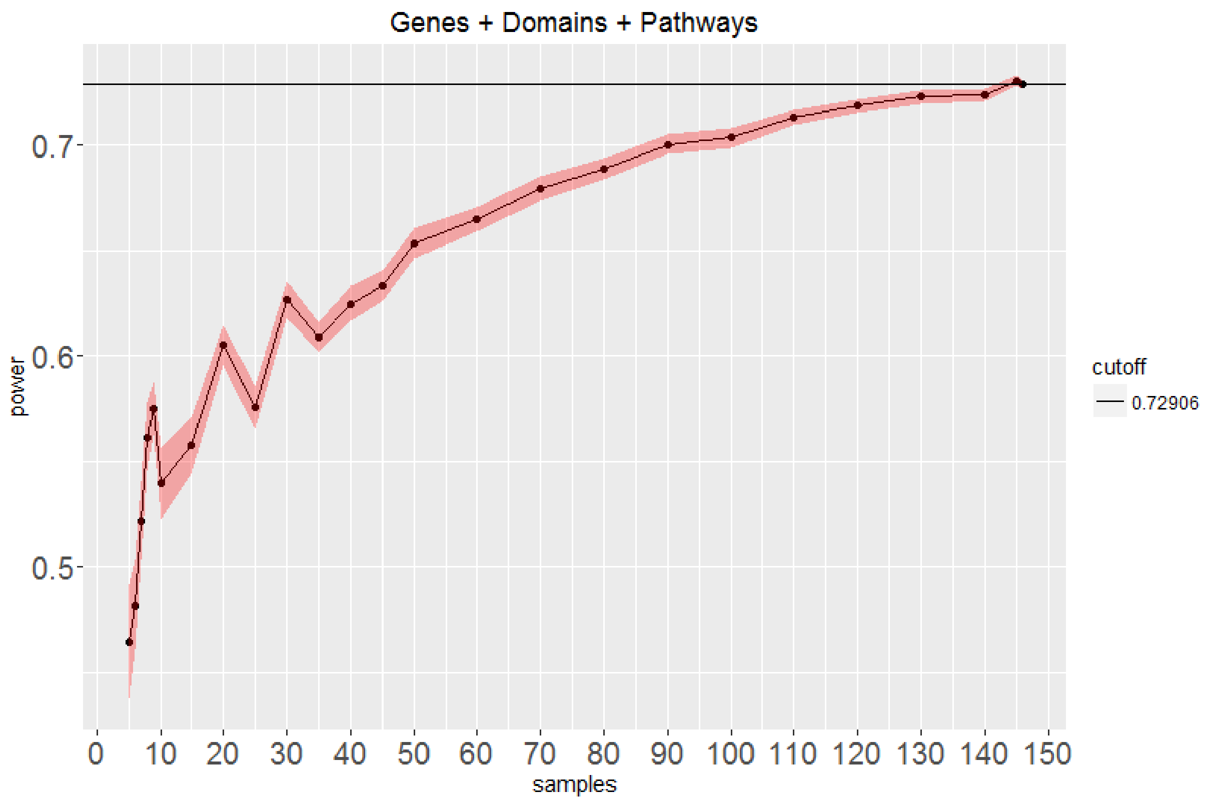
Genes + Domains + Pathways
power
0.7
0.6
0.5
samples
0
10
20
30
40
50
60
70
80
90
100
110
120
130
140
150
cutoff
0.72906

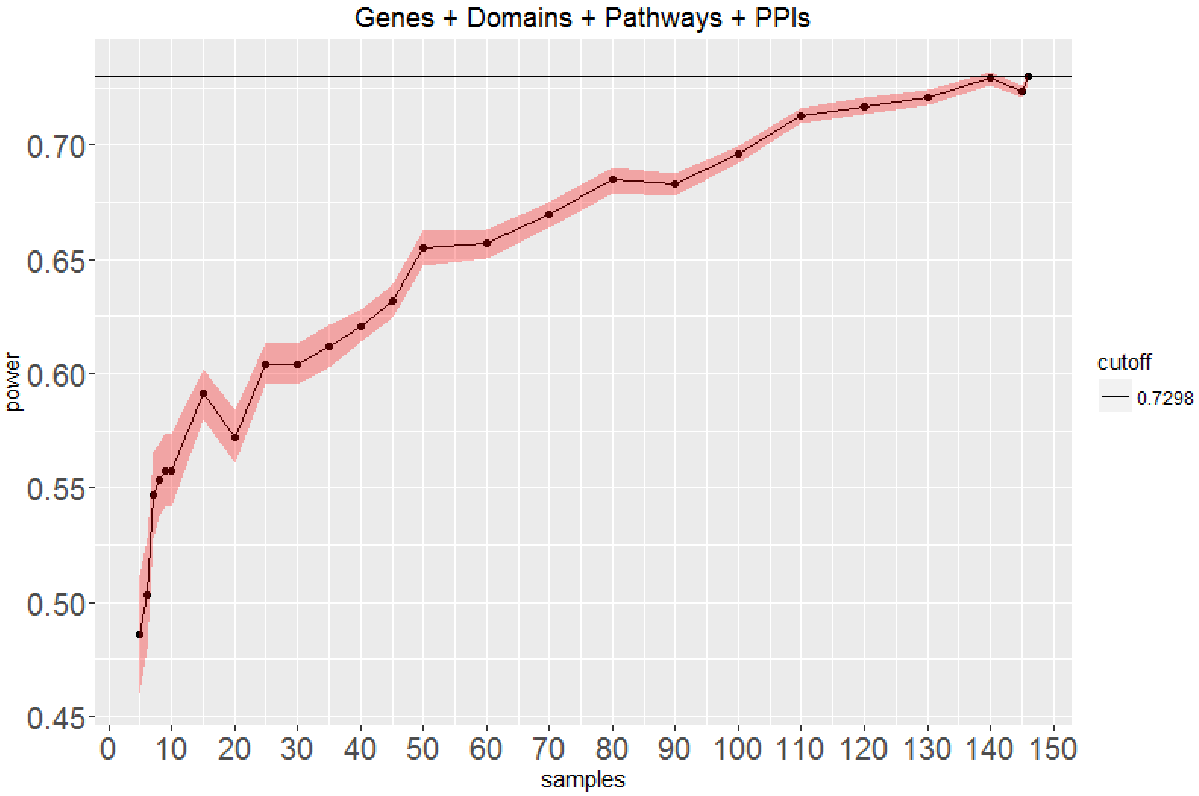
Genes + Domains + Pathways + PPIs
0.70
0.65
0.60
0.55
0.50
0.45
power
0 10 20 30 40 50 60 70 80 90 100 110 120 130 140 150
samples
cutoff
0.7298

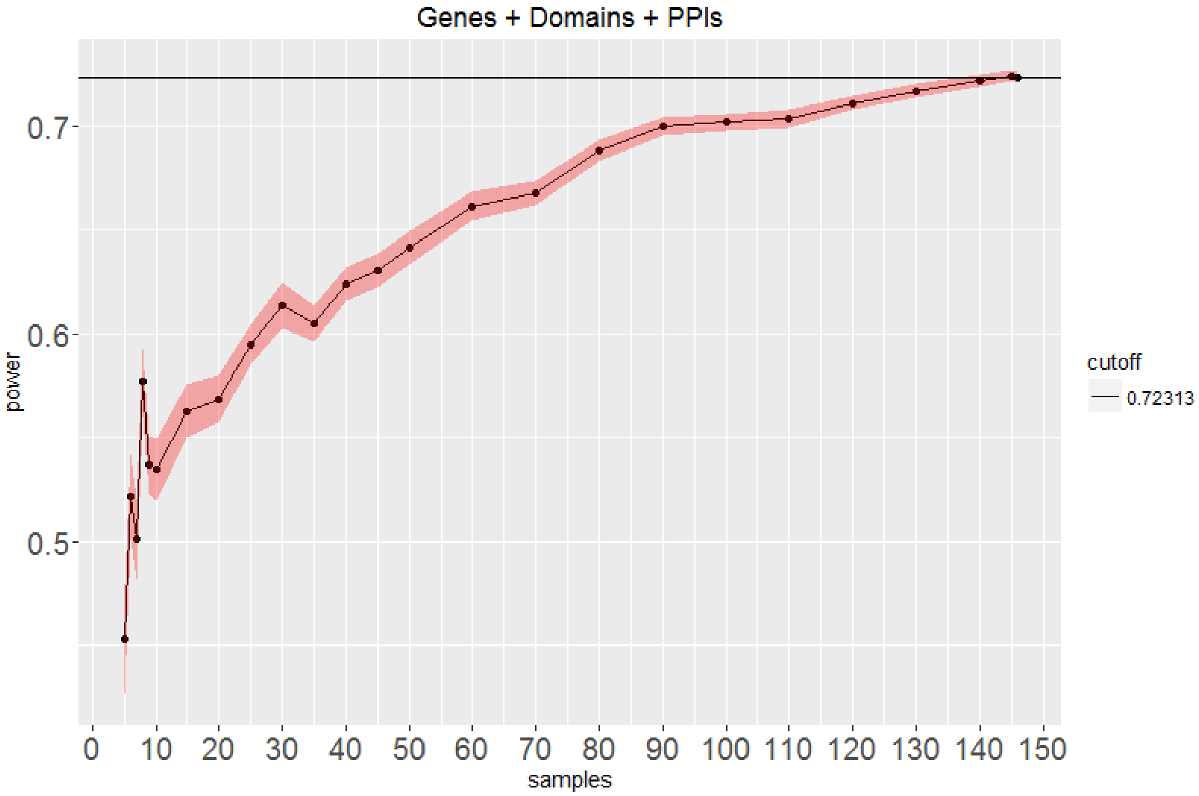
Genes + Domains + PPIs
power
0.5
0.6
0.7
samples
0
10
20
30
40
50
60
70
80
90
100
110
120
130
140
150
cutoff
0.72313

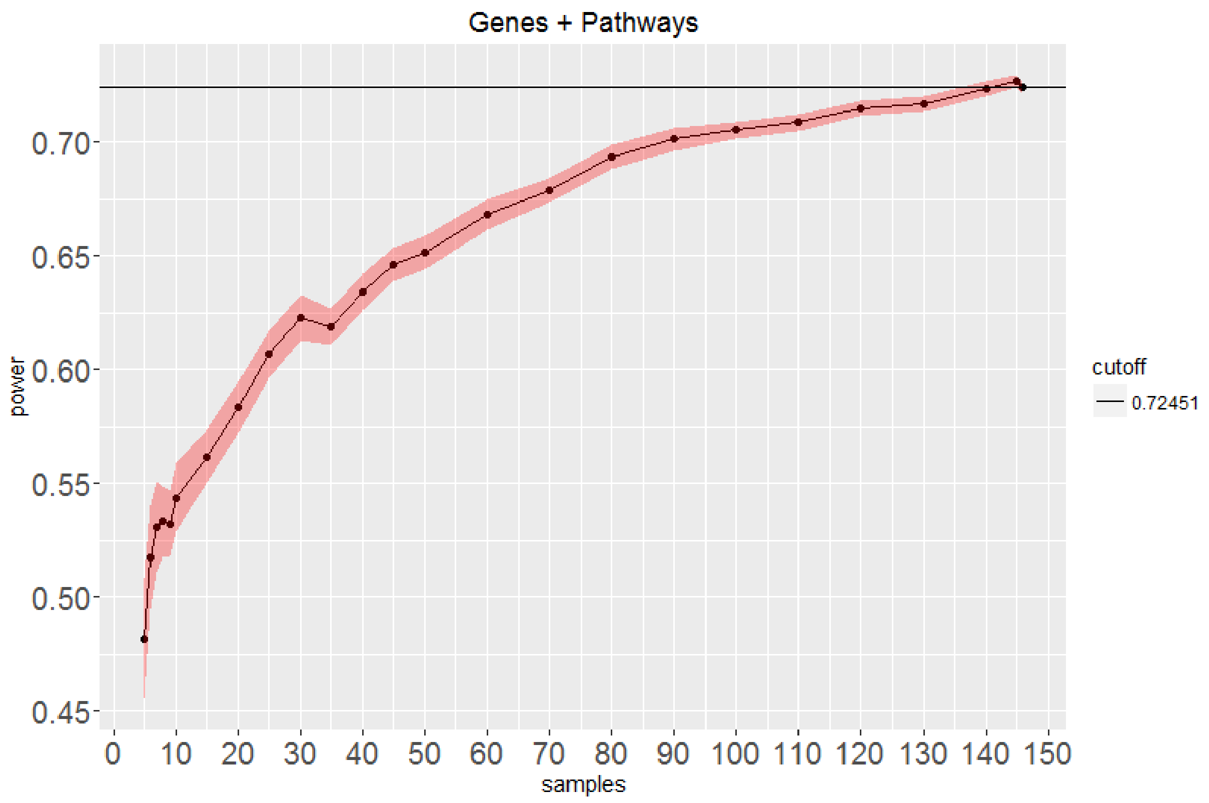

Genes + Pathways
power
0.70
0.65
0.60
0.55
0.50
0.45
0 10 20 30 40 50 60 70 80 90 100 110 120 130 140 150
samples
cutoff
0.72451

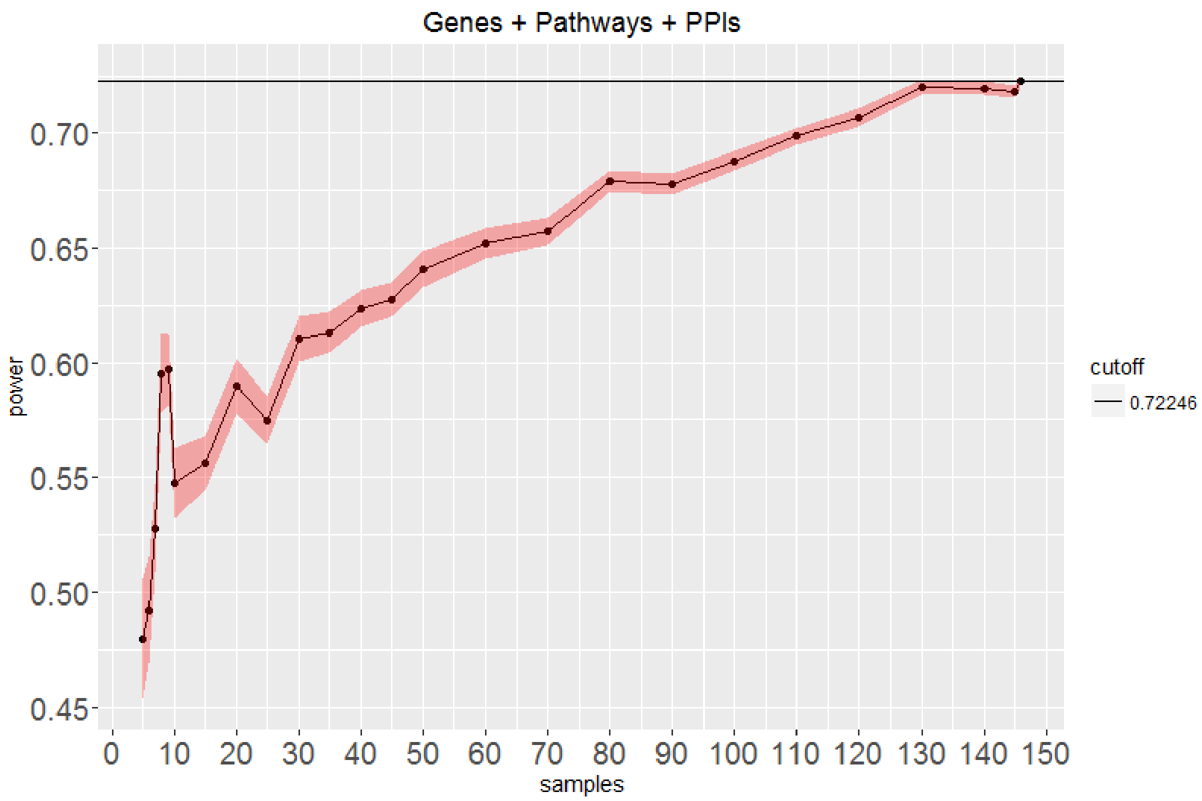
Genes + Pathways + PPIs
power
samples
cutoff
0.72246
0.45
0.50
0.55
0.60
0.65
0.70
0
10
20
30
40
50
60
70
80
90
100
110
120
130
140
150

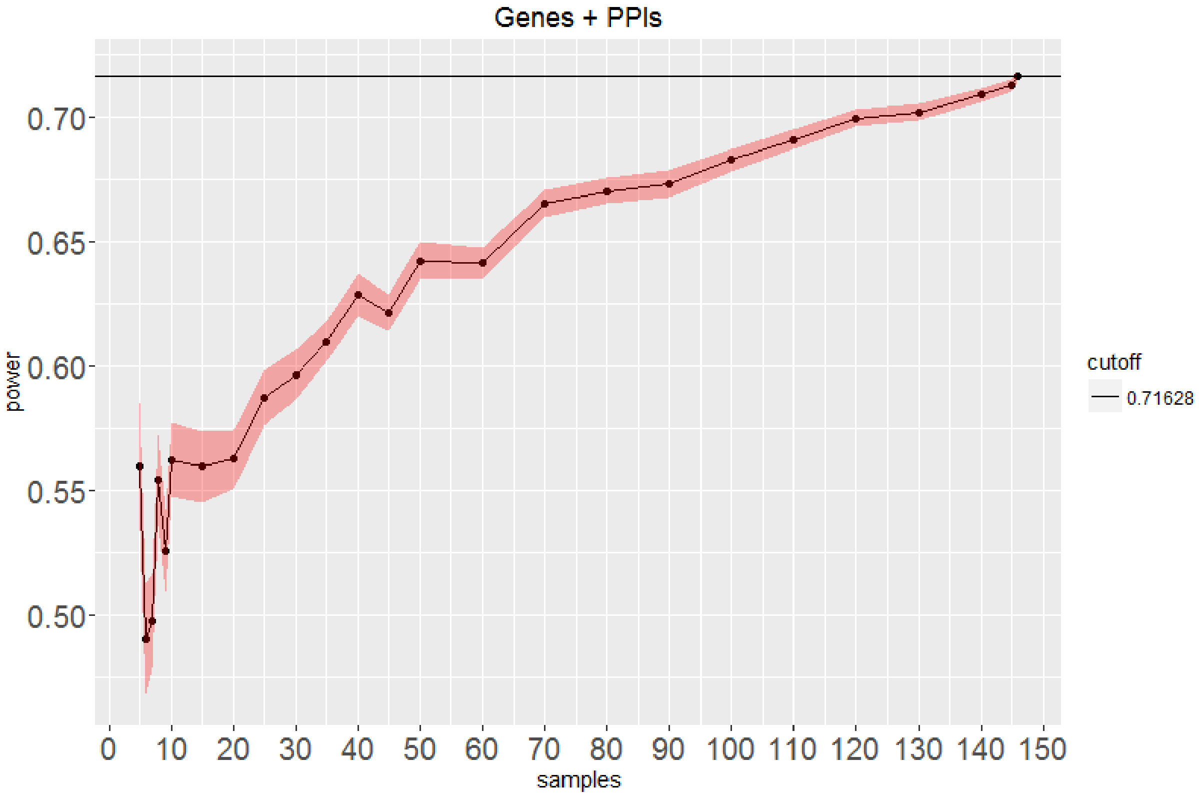
Genes + PPIs
power
0.70
0.65
0.60
0.55
0.50
0 10 20 30 40 50 60 70 80 90 100 110 120 130 140 150
samples
cutoff
0.71628

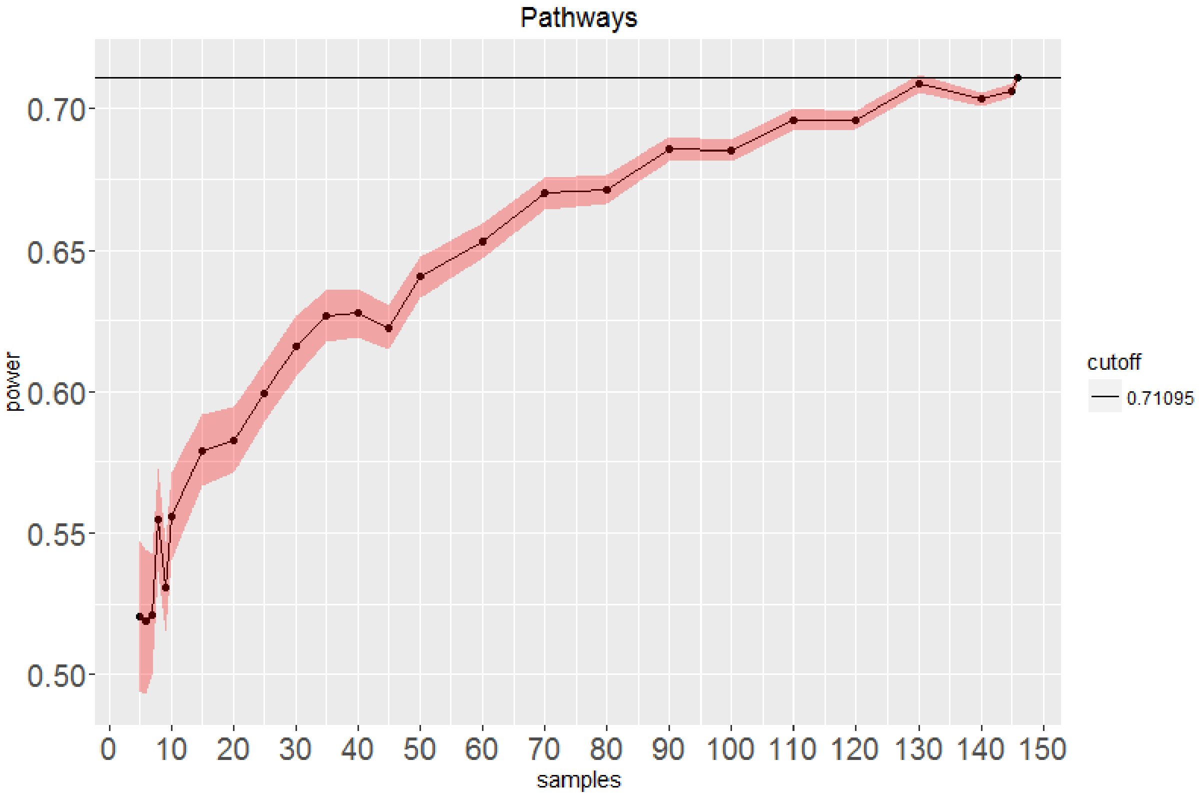
Pathways
power
0.70
0.65
0.60
0.55
0.50
0
10
20
30
40
50
60
70
80
90
100
110
120
130
140
150
samples
cutoff
0.71095

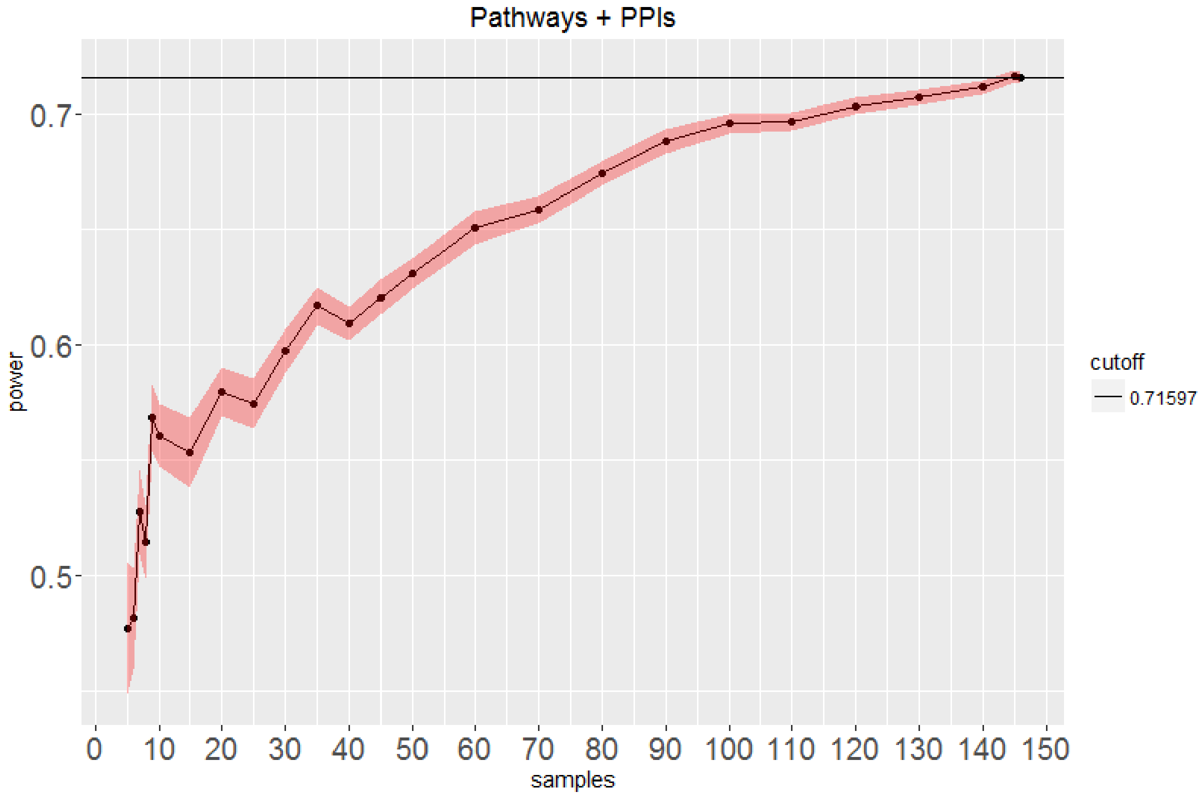
Pathways + PPIs
power
0.7
0.6
0.5
samples
0
10
20
30
40
50
60
70
80
90
100
110
120
130
140
150
cutoff
0.71597

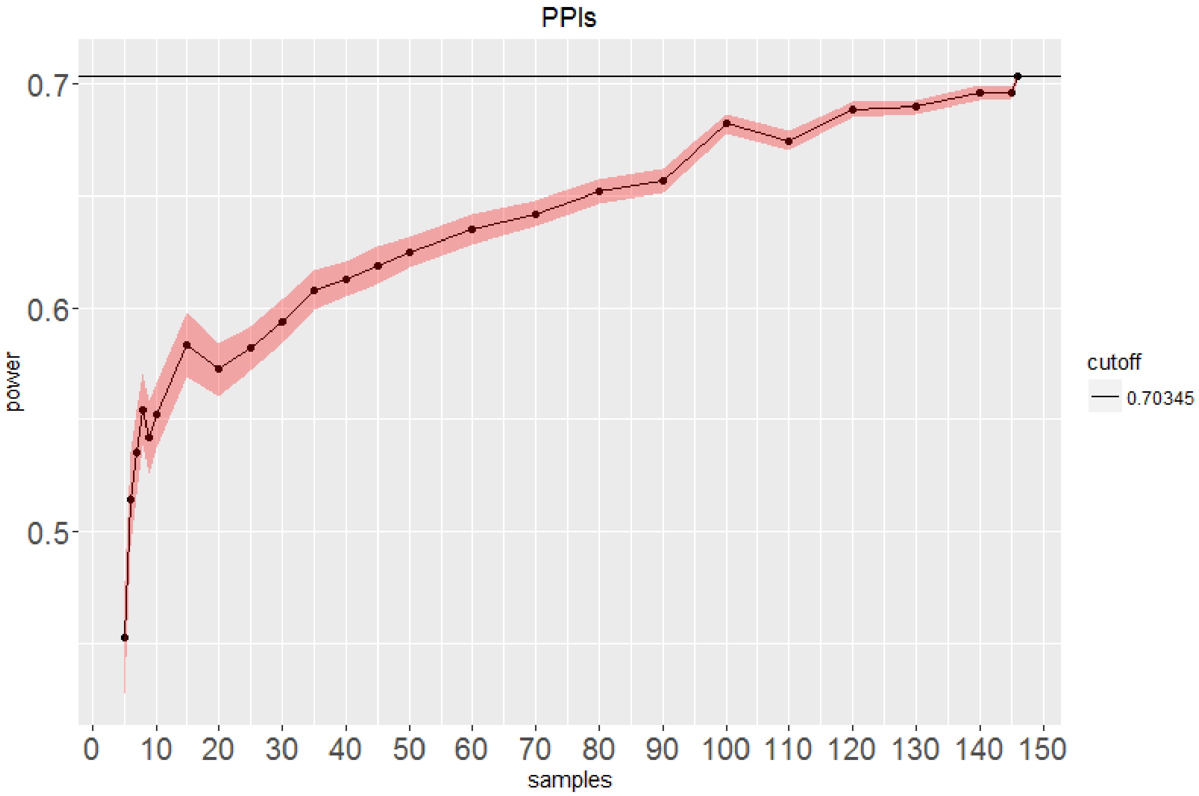

PPIs
power
samples
cutoff
0.70345
0.5
0.6
0.7
0
10
20
30
40
50
60
70
80
90
100
110
120
130
140
150

Sample size analysis, univariate

The effect of sample size on univariate analysis was calculated with simulations. The following procedure was applied to each feature:

1. Simulate new data based on the variable's distribution observed in cases and controls (mean, standard deviation, number of observations) and estimate the Cohen's effect size as follows:

   abs(mean group 1 – mean group 2) / standard deviation (pooled)

   where *abs* is the absolute value.

2. Perform a Wilcoxon Rank Sum test to test for the presence of statistically significant differences in terms of the simulated variable's distribution between class = 1 and class = 0 and collect the deriving p-values.

The simulating-and-testing procedure was repeated 1000 times for each variable and the frequency of statistically significant differences (i.e., the number of times the p-value was < 0.05 divided by the number of simulations performed) estimated (statistical power).

Results are reported in the Supplementary Figure 16, describing variations in terms of statistical power distribution obtained from simulations as function of the effect size observed from data, given the number of cases and controls analysed and assuming a significance threshold of $p < 0.05$.

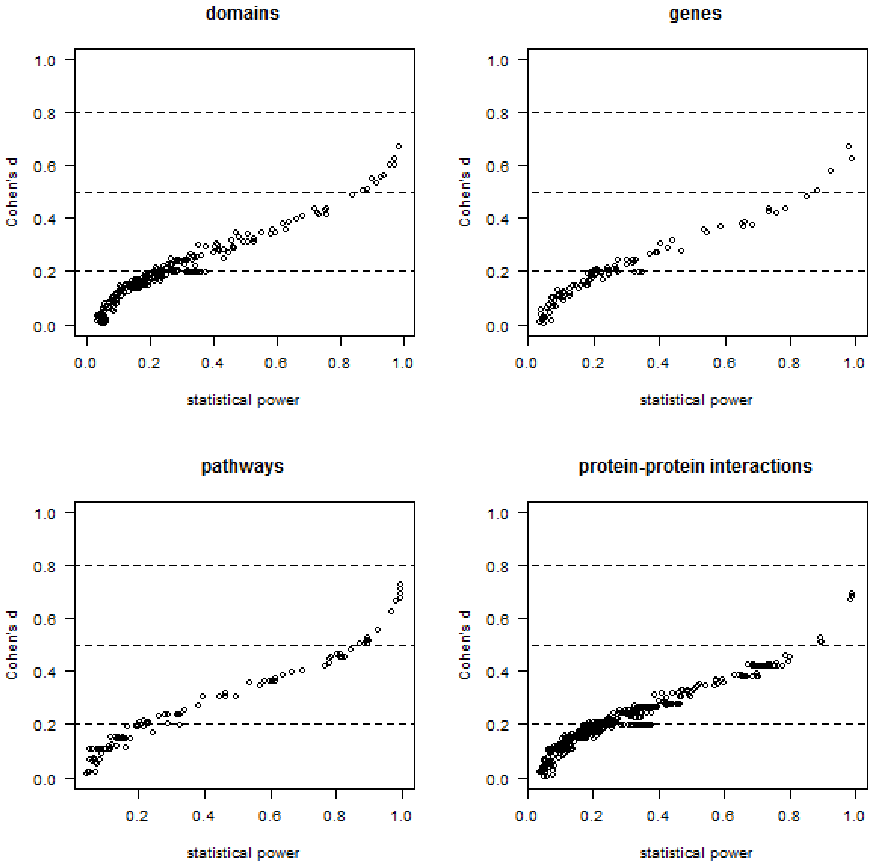

Figure S16. Scatterplots reporting variations in terms of statistical power (x-axis) as function of the observed effect size, quantified in terms of Cohen's d (y-axis) for each dataset. The horizontal dashed lines highlight the thresholds corresponding to d values of 0.2, 0.5, and 0.8, representing small, medium, and large effect sizes respectively, according to Rosenthal and Rosnow (1984, p.361).

Results (Figure S16 and Table S1) show that the study is sufficiently powered (> 80%) to detect intermediate to large effect sizes (0.5 < d < 0.8, given the number of cases and controls analysed and assuming a significance threshold of $p < 0.05$).

**Cohen’s d**	**Domains**	**Genes**	**Pathways**	**Protein-protein interactions**
d < 0.2	0	0	0	0
0.2 < d < 0.5	1.27	2.78	15.38	0.49
0.5 < d < 0.8	100	100	100	100

Table S1. Frequency (%) of features reaching statistical power > 80% by effect size ranges based on simulations.

As shown in Supplementary Figure 16, the required Cohen's d level for achieving 0.8 power is ~0.5. Cohen's d has been measured fore each statistically significant ROI, and found in the range [0.57-0.77].

SCN1A Cohen's d estimate: 0.7045188

CHRNB2 Cohen's d estimate: 0.6538497

CACNA1G Cohen's d estimate: 0.5975064

IPR001696 Cohen's d estimate: 0.6229835

IPR010526 Cohen's d estimate: 0.6272407

IPR005821 Cohen's d estimate: 0.7059557

IPR001098 Cohen's d estimate: 0.5810361

hsa04930 Cohen's d estimate: 0.7543276

hsa04728 Cohen's d estimate: 0.7756596

hsa04725 Cohen's d estimate: 0.6528608

hsa04919 Cohen's d estimate: 0.572608

hsa04976 Cohen's d estimate: 0.574127

hsa04911 Cohen's d estimate: 0.6966038

hsa05033 Cohen's d estimate: 0.7097573

hsa04020 Cohen's d estimate: 0.7296446

UBC_ppi Cohen's d estimate: 0.7167586

SNTA1_ppi Cohen's d estimate: 0.7288087

PSEN1_ppi Cohen's d estimate: 0.7045188

Supplementary References

Jacob Cohen (1988). Statistical Power Analysis for the Behavioral Sciences (second ed.). Lawrence Erlbaum Associates

Rosenthal, R. and R.L. Rosnow (1984), Essentials of Behavioral Research: Methods and Data Analysis. New York: McGraw-Hill.
